# Education of nursing profession amid COVID-19 Pandemic: A qualitative study

**DOI:** 10.30476/JAMP.2021.90779.1422

**Published:** 2022-01

**Authors:** MARYAM TOLYAT, SEYYED ABOLFAZL VAGHARSEYYEDIN, MARYAM NAKHAEI

**Affiliations:** 1 Faculty of Paramedical, Birjand University of Medical Sciences, Birjand, Iran

**Keywords:** Education, Nursing, Coronavirus, COVID-19

## Abstract

**Introduction:**

COVID-19 pandemic is the main challenge with which the education systems in the world have ever faced. Furthermore, nursing students and nurse educators have faced the challenges in the teaching-learning process. This study aimed to explain the experiences of nursing education amid COVID-19 pandemic.

**Methods:**

This research was conducted in 2020-2021 through qualitative content analysis approach. Participants were selected from nursing schools using purposive sampling. Data were collected using in‐depth, unstructured, and face‐to‐face interviews. The interviews were digitally recorded and transcribed verbatim. The conventional content analysis method was used to analyze the data.

**Results:**

232 codes were generated which were grouped into four main categories, namely mandatory change in nursing education, change of training priorities of nurse educators, insufficient clinical competence, and opportunities during coronavirus outbreak.

**Conclusions:**

COVID-19 prevalence caused many unprecedented changes in nursing education. Such changes have brought about opportunities and challenges in nursing education.

## Introduction

The COVID-19 pandemic has become a major crisis in global health. This pandemic deeply changed human behavior on different levels ( 1 , 2 ). Depending on the prevalence and severity, countries had to apply different scenarios such as adherence to health protocols, social distancing, lockdown and restrictions on domestic and foreign travels, and the closure of places with mass gatherings ( 3 , 4 ). 

 In the education system, the nursing education had to change their routine methods and adjust their education based on health protocols ( 5 ). These changes required the education to move toward e-learning in nursing required in implementation of student-centered education, and evaluation strategies have been modified ( 6 ). On the other hand, more than half of the nursing courses in the nursing curriculum are held clinically and practically ( 7 ).

Nursing students and nurse educators also faced challenges in the teaching-learning process, especially students' presence in clinical wards, knowledge-based assessment, skills and attitudes, quality management in health care, and patient safety ( 8 ). The challenge of providing clinical education has increased the use of simulation, distance health, and virtual reality applied by nursing faculty members in different countries, and access to these resources has been another challenge in nursing schools ( 9 ). 

Various studies have been conducted on the impact of pandemics on medical education ( 4 , 10 , 11 ) In 2009, a study entitled” the challenges of continuing medical education in a pandemic era” was conducted by Lim. Pandemic outbreaks had unique challenges to medical educationists, i.e. to decide whether the situation merits school closure and how to continue with clinical training. Videotaped vignettes and audiotaped recordings or simulators, webcasting, and online chat rooms, were successfully adopted by medical schools during the SARS outbreak ( 12 ). 

In Redinger’s study, adapting to a virtual classroom, embracing informal teaching, and supporting trainee mental health were the strategies that were implemented in the SARS pandemic in medical education ( 13 ).

Iran started to use various methods of virtual education as an alternative to in-person training as soon as possible ( 2 ). To prevent the spread of disease and reduce the high workload in hospital wards, many universities of medical sciences asked the students to leave academic and clinical settings and directed them to virtual education ( 14 , 15 ). Like other countries, regarding the increased need of the healthcare system to nurses and graduation of the senior students in the absence of disease crisis, their clinical education was held in different departments by reducing the training hours ( 9 ), and a number of students were graduated.

Although a year has passed since the outbreak of COVID-19, and nursing education in Iran, as in other countries, has undergone changes, we have limited knowledge of the experiences of nursing education. A qualitative study can help to shed light on the experiences of nurse educators and students in this area. Therefore, a qualitative study was conducted to explore the experiences of nurse educators and nursing students in Iran.

## Methods

This qualitative study was conducted using conventional content analysis approach. The aim of this study was to explore the experiences of nursing education amid the COVID-19 Pandemic.

### Setting

This study was conducted in Nursing and Midwifery College affiliated to Birjand University of Medical Sciences, Birjand, Iran. Participants were 12 nurse educators and 7 nursing students in this college. 

### Participants

Interviewees were selected purposefully from nurse educators and nursing students in the nursing faculties affiliated with Birjand University of Medical Sciences.

### Data collection

Data were collected via in‐depth, unstructured, and face‐to‐face interviews. The time and place of interviews were selected as agreed with the participants. Interviews were recorded by digital audio recording. The interview was initiated with an open-ended primary question such as: “Could you please explain your experience of nursing education during the COVID-19 pandemic?” They were continued with probing questions such as “Would you please explain more?” Interviews lasted approximately 60 to 90 minutes. All interviews were audio-recorded with permission of the participants and transcribed verbatim by the researcher. Data collection and analysis were done from August 2020 to January 2021. The data collection process continued until the data were saturated and no new categories were emerged from the data. Finally, 19 nurse educators and nursing students were purposively selected for interview.

### Data analysis

Data were analyzed using Graneheim and Lundman's content analysis method. All interviews were listened to several times to obtain a sense of the whole, and then they were transcribed verbatim. The meaning units in quotations were extracted and labeled with a code. The various codes were compared based on differences and similarities. The codes similar in meaning were sorted into subcategories. This was done by the first author. Subsequently, subcategories were compared and combined with each other to form the main categories. To analyze the data, MAXQDA software 10 was used.

### Rigor

Lincoln and Guba’s criteria were used to ensure the validity and reliability of the data ( 16 ). To ensure credibility, the researcher had a long and deep involvement with the subject and data as well as member checking in such a way the transcripts were made available to participants along with the initial codes. In addition, the credibility of the data was enhanced using different data collection methods (interviews, observations, and memos). 

 The data dependability was assessed, using both peer and member checking. The primary findings of the study along with the preliminary codes and categories were shared with the participants and their opinions were received (member check). Some parts of the data were analyzed by other colleagues who were not involved in the study (peer check). Confirmability was ensured through expert check. The codes and categories were repeatedly monitored and confirmed by supervisors (expert check). Finally, by providing a comprehensive description of the topics, participants, data collection and analysis procedures, and limitations of the study, we made an attempt to create transferability, so that other researchers may clearly follow the research process taken by the researchers.

### Ethical considerations

The study was approved by the Ethics Committee of Birjand University of Medical Sciences, with the ethical code of IR.bums.REC.1397.386. At the beginning of the interviews, the aim of the study was explained to the participants. Participants signed written consent forms and allowed us to record their voices during the interviews. They were assured that they could leave the study at any point and that their identities would be kept confidential by researchers.

## Results

Ninety nurse educators and nursing students participated in the study. Eleven participants were female and eight were male. Twelve participants were nurse educators, and the others were nursing students. The mean age of the nurse educators was 42.05±8/9 years, and that of nursing students was 22.5±3.03 years. During the process of data analysis, 232 codes were generated and categorized into 11 subcategories and for main categories. Regarding the exploration experiences of nursing education during the COVID-19 outbreak, 4 categories and 11 subcategories were extracted ( Table 1).

**Table 1:** Categories and subcategories of experience of the nursing education

Meaning unit	code	Subcategory	Category
• In the beginning, the classes were held offline. We recorded audio on the slides and uploaded them in the system for students to see.	• Compulsory termination of face-to-face training in theoretical and clinical courses	Shift to virtual education	Mandatory change in the nursing education
• To teach clinical skills, we performed the techniques ourselves, videotaped them, and uploaded the videos.	• facing the challenges of e-learning
	• Replacing virtual education with face-to-face training in theoretical and practical courses
• Education at the university was stopped for some time due to the corona virus outbreak.	• Unpredictability of internships due to the peak of the disease	Unpredictable training conditions
• Due to the corona virus pandemic, our programs have been completely disrupted; we continue with the internship for two weeks but then it stops for a while; we do not know what we are going to do next.	• Reduce the forecast of educational and protective facilities for students and teachers	
• I upload the educational content in the system and do not care if the student reads it or not.	• Upload educational content to the student to study	Changing from a teacher-centered to a student-centered education	Change of the training priorities of the nurse educators
	• Reduce the sense of responsibility for student learning		
• During the internship, if the patient was suspicious or positive of corona virus disease, we never approached the patient, although the cases for training purposes were few.	• Optional nursing care for students in internships	Conservative attitude towards the disease
	• Student fear of entering the patient's room	
• Our instructor did not allow us to perform high-risk techniques, such as suctioning the patient.	• Transfer of clinical education to classrooms due to less clinical exposure	
	• Non-interference of the student in performing high-risk techniques in the internship by the instructor	
• Now everyone just wants the internships to end. They shortened the three weeks of internships to one week.	• Less attention to student learning in clinical education than before	Inattention to the educational process
• Nobody in the internships checks who comes, who fails to come, if the student learns or not.	• Lack of attention to student attendance at internships
	• Underestimation of internship by instructor and student
• I am now in the eighth semester and about to graduate, but I have not practiced many of the skills. I feel I rarely know anything.	• Feeling less qualified due to reduced internship hours	Insufficient self-esteem in acquiring clinical competency	Insufficient clinical competence
	• Feel less learning in internships than before		
• When I graduate, I am expected to work as a nurse, but I do not think I can be a good nurse. The corona virus struck us hard.	• Failure to do so skills due to the reduction in the number and variety of patients		
• Our communication with the patient has become too limited. We used to go over the patient and talk to him/her; now we only go for what is really needed.	• Decreased communication with the patient compared to before	Weakness in providing holistic nursing care	
• The student-patient relationship is very superficial. The student is afraid to approach the patient.	• Superficial performance of nursing care by the student due to fear		
• I was not familiar with and did not use e-learning before the corona virus outbreak. It did well to us, and we came to learn these methods.	• Familiarity with e-learning methods	Ability to use virtual education	Opportunities in the COVID-19 pandemic
	• Application of e-learning methods in nursing education		
• These videos that we have prepared can be stored as information in the system so that the student can watch them whenever s/he has a problem.		
• The corona virus was good in one way; it highlighted the important role of the nurse in the treatment team.	• Depicting the role of nurses in the corona pandemic in the media	The importance of the nurse role in the healthcare team
	• Direct exposure of people to nurses	
• Previously, when we went to internships, there were no vacant classes. Many times we held our classes in the corridor. Now the classes are all empty.	Easy access to patients and files due to the decrease in internship students compared to before	Easier access to educational facilities
• Because not all students are present in the wards, the wards are now secluded, and access to patients and their files is much easier.	• Easier access to classrooms than before	
• Formerly, no one observed standard precautions in the wards, but now you see that doctors, nurses, students, and instructors all comply with them carefully.	• More emphasis on standard precautions by the instructor	More sensitivity to the observance of health protocols
	More standard precautions by instructors and students compared to before the pandemic	

### 1. Mandatory change in the nursing education

Mandatory change in the nursing education was one of the experiences of the nursing students and educators. The mandatory change was experienced in the form of shifting to virtual education and the unpredictable training conditions. Nurse educators mentioned many challenges during virtual education such as the managers’ lack of attention to the problems of virtual education, structural problems in the virtual education system, time-consuming preparation of educational content, and less teacher-student interaction.

One of the educators said:

*“Holding classes online was very hard and time consuming. In addition, the system was problematic. The Internet was also weak and sometimes became disconnected. The content loading was very time-consuming and sometimes it took 4-5 hours. There were frequent errors in the middle of uploading.”*

The unpredictable training condition was another subcategory of mandatory change in the nursing education. Owing to the fact that there is no suitable prognosis for this disease, educational conditions are not predictable, and it is not possible to provide a fixed planning to continue the training process.

One of the students stated:

*“There is no arrangement in our programs in this unstable situation. The training may be stopped for one week. Then, we again start training, but it is stopped and we have to stay at home for two months. We are really tired of this situation.”*

Regarding the mandatory change in the nursing education, the authorities did not anticipate the need for more personal protective equipment. As students and educators acknowledged, there is a greater need for personal protective equipment during training in the current situation, which has created additional concerns for educators and students and passed them round to different hospitals and the educational system to provide the protective equipment.

The experience of the students is as follows:

*“When we were being trained, we asked for personal protective equipment. They passed us round to the hospitals and the faculty. We were stuck in this situation.”*

### 2. Change of the training priorities of the nurse educators

This was another experience of nurse educators and nursing students in nursing education during the COVID-19 outbreak. Changing from a teacher-centered to a student-centered education, a conservative attitude towards the disease, and lack of attention to the educational process were the experiences expressed by educators and students.

They experienced changing from a teacher-centered to a student-centered context. Students were responsible for their learning and educators did not take responsibility as before.

One of the educators said:

*“I upload the educational content for the students. Now it depends onthe student whether he/she wants to study or not.”*

The overemphasis on personal protection in clinical education was another priority of the educators and students in clinical settings. To protect themselves from the disease, they tried to spend the least time in the clinical setting and only did clinical procedures, and sometimes they avoided entering the patients' rooms and devoted most of their training time to theoretical classes. 

One of the nursing students explained his/her experience as follows:

*“The educator used to appoint a patient for us during the training, and we had to do everything for the patient. In addition, she/he did not allow us to contact with a patient suspected of the COVID-19. Now, we are free to choose among patients and it is up to us whether to contact with COVID-19 patients or not.”*

The educators’ lowering of personal standards for teaching was another experience expressed by the nursing students during the training. They mentioned that their educators were not serious in the teaching process and their goal was only to pass the time. The educator does not pay attention to the presence of the student during training and does not observe educational regulations. 

One of the students explained as follows:

*“Educators used to explain educational materials with more details, but now they explain them briefly. They even do not ask us to present research or conference”.*

### 3. Insufficient clinical competence

Most of the nurse educators and nursing students mentioned that students did not acquire the necessary clinical competence in nursing education in the current situation and considered insufficient clinical competence as an insufficient self-esteem in acquiring clinical competence and a weakness in providing holistic nursing care.

Most of the nurse educators mentioned that the number and variety of patients admitted to the elective wards had reduced due to the COVID-19 outbreak, and they did not gain sufficient experience with different cases. In addition, the reduction of training hours is another important factor in reducing the students' clinical competencies. This change caused poor clinical competency of students.

One of the nursing students explained his experience as follows:

*“Now, the level of learning in the training is very poor and inefficient compared with before COVID-19. We do not observe many cases. I have passed the emergency department training, but I am worried about the skills that I have not practiced even once.”*

Nurse educators and nursing students pointed out that nursing students were providing poor nursing care to patients. Due to the fear of COVID-19, students try to have minimal contact with the patient and only go to the patient's room to perform nursing skills. They do not pay attention to providing nursing care for the patient psychologically.

One of the nursing students explained as follows:

*“We are a bit afraid in the training. We used to communicate with the patient easily and do what we really can for the patient. We think our care has become superficial. I used to talk with the patient when giving him/her medications or doing a procedure, but we try not to contact with the patient in these circumstances.”*

### 4. Opportunities in the COVID-19 pandemic

Participants mentioned that regarding various problems and challenges during the disease, the current situation also created opportunities in nursing, including the ability to use virtual education, the importance of the nurse role in the healthcare team, easier access to educational facilities and more sensitivity to the observance of health protocols.

The ability of educators to use virtual education was one of the opportunities experienced by nurse educators. Nursing education was done traditionally before coronavirus and educators rarely used virtual education because they were not familiar with e-learning methods. Therefore, with the mandatory change in the teaching process, educators became familiar with these methods, and they even can present part of their educational content virtually after coronavirus pandemic. One of the educators stated:

*“Coronavirus made us familiar with e-learning and helped us hold at least some of the classes online in later semesters.”*

The importance of the role of nurses in the healthcare team was one of the opportunities created in the COVID-19 conditions. Radio, television, and other social media portrayed the role of the nurse in the treatment of coronavirus disease, which led to a positive attitude of students and educators towards the nursing profession. One student expressed:

*“Corona was really good and proved something to everyone. Some people believe that nurses are just responsible for injecting ampoules and giving medications and the doctors do the rest, but corona showed the role of nursing clearly.”*

Easier access to educational facilities was another opportunity experienced by nursing students. They previously faced insufficient educational facilities in the clinical setting due to student overcrowding in various fields and even had difficulty in holding classes. In the current situation, due to the division of students and absence of many groups in the clinical settings, they have easier access to educational facilities such as classrooms and patients’ medical records. One nursing student explained as follows:

*"Owing to the fact that the training did not overlap with different groups, we were comfortable during the training. Medical students and other students were not present; the wards were not crowded, so we could use classes whenever we wanted. Most of the classes were empty.”*

Another opportunity experienced by educators and students was greater sensitivity and adherence to health protocols, such as washing hands, using masks and shields, and maintaining social distance.

One nurse educator explained her experience as follows: 

*“Students, educators and staff are more sensitive to adhere to the protocols in these situations. I urge the students to adhere to hand hygiene and wear masks and shields if necessary. Students are also very sensitive and adhere to them.”*

## Discussion

This study aimed to describe the experiences of nurse educators and nursing students in nursing education during the COVID-19 outbreak. Four main categories were extracted from the data collected in this study, including mandatory change in the nursing education, change of the training priorities of the nurse educators, insufficient clinical competency, and opportunities in COVID-19 pandemic.

The category of mandatory change in the nursing education included two subcategories of shift to virtual education and unpredictable training conditions. Participants in this study pointed to problems such as lack of infrastructure, reduced readiness of educators and students to use e-learning, time-consuming preparation of educational content, less teacher-student interaction, and managers’ lack of attention to e-learning problems. Other studies have also mentioned such challenges ( 2 , 10 , 17 - 19 )

The unpredictable training conditions were another study finding. Iran, like other countries, had to close educational centers and stop the educational process in person at the beginning of the COVID-19 outbreak to deal with the consequences of the disease. Therefore, in-person education gave way to online education because the continuation of in-person education was unpredictable ( 7 , 20 ). Thus, it seems it is necessary that the managers upgrade and develop virtual education and encourage the use of up-to-date technologies.

 According to the previous category, participants stated that by changing the teaching method, educational priorities also changed. In fact, change of the educational priorities of nurse educators was another experience of nurse educators and nursing students in the COVID-19 pandemic. In the present study, overemphasis on personal protection, educators’ lowering of personal standards for teaching, and reduced attention of students and educators to learning clinical skills were described. In the present study, like other studies, students and educators were highly stressed due to coronavirus disease ( 21 ). Studies reported that students' stress was much higher than normal. In the present study, students’ high stress caused excessive observance of health protocols to protect themselves and their families. Aslan showed that 68.1% of the students were worried about their infection ( 22 ). Therefore, nurse educators should pay special attention to students' stress ( 23 ).

Nursing educators believed that changes in nursing education led to insufficient clinical competence in students. Obviously, they believe that nursing students need to be constantly exposed to clinical procedures and settings to gain clinical competencies. Although the effect of the prevalence of COVID-19 on students' clinical competency has not been objectively investigated ( 24 ), we can logically assume that students have not yet acquired the necessary clinical competencies, and the training conditions have reduced their clinical competencies ( 25 ). Due to the COVID-19 epidemic, many students have lost the chance to learn clinically because their clinical skills and professional identity can only be acquired in a real clinical setting ( 7 ). Sanago et al. have mentioned simulators as a solution for clinical education of students in the coronavirus pandemic ( 26 ). The World Health Organization also emphasizes that simulators in nursing and midwifery education provide benefits for students and patients and can train safer and faster interventions to caregivers that comply with international recommendations, so they improve the overall quality of care ( 27 ). Given that clinical training does not have the necessary qualification and quality, and educators and students are concerned about the clinical competency, the use of alternative solutions, including simulators in clinical education can help the students acquire appropriate clinical competency. 

 Although the COVID-19 outbreak created various problems and challenges, it also created opportunities in nursing education, including the educators’ ability to use virtual education, the importance of the role of nurse in the healthcare team, easier access to educational facilities and greater sensitivity to adhere to health protocols.

The ability of educators to use e-learning was one of the opportunities experienced by nurse educators ( 28 ). Similar to other studies, with change in the teaching process, nursing educators became acquainted with these methods ( 19 , 25 ). Various studies reported flexibility, opportunities created for learning, use of different e-learning methods, online applications, interaction with the world around, online meetings, online assignments, storage of content in the Web, online counseling, and the effective use of electronic media to share information as the online education opportunities in the COVID-19 pandemic ( 11 , 19 ).

The importance of the nurses’ role in the healthcare team was another finding. For example, radio, television, and other social media portrayed the role of nurses in the treatment of COVID-19 patients, which led to a positive attitude of students and educators towards the nursing profession. In other studies, most of the nurses reported that public perceptions of their profession changed in the COVID-19 pandemic. In addition, the socially formed image made the nurses feel valued ( 11 , 29 ).

The present study mentioned educators and nursing students’ easier access to educational facilities. One of the main challenges for nursing students in clinical education is insufficient educational space ( 30 ) due to student overcrowding. Student overcrowding in departments is due to reduced coordination in planning clinical education for students in different fields and high number of students in colleges. Therefore, in the current situation, students have easier access to educational facilities such as classrooms, patients, and their medical records due to the reduction in the number of students in the departments, and the absence of some students in other fields in the clinical setting.

Another opportunity experienced by nurse educators and nursing students in coronavirus pandemic was greater sensitivity to adhere to health protocols. In the present study, as in other studies, many students, educators, and staff adhered to health protocols such as washing hands, wearing masks and shields, and maintaining social distance with greater sensitivity and accuracy to protect themselves and patients ( 22 , 31 ). The public attitude towards health protocols changed in the COVID-19 pandemic, which can be an opportunity in better adherence to standard precautions and health protocols after the pandemic ( 32 ).

## Limitation

In terms of limitation, it can be noted that due to the emergence of the corona disease, the number of published research articles was limited at the time of the study. This limitation was clearer in domestic studies. Therefore, it is suggested that more quantitative and qualitative studies should be conducted on the use of e-learning in the Corona course and the challenges ahead in nursing education.

## Conclusion

The findings of this study showed that the COVID-19 outbreak deeply affected nursing education. Educational program shifted from face-to-face to virtual education. However, this change created challenges and opportunities. Challenges and opportunities can lead the nursing education to innovation. They can also develop new approaches to implementing the content and competencies tailored to evolving social needs. Therefore, due to the change in the learning environment caused by the epidemic, the integration of technology in education is not a choice but a need for all authorities who themselves need flexibility, creativity and innovation. What we do know is that nursing students need to acquire clinical competency. Owing to the fact that acquiring clinical competency in nursing education is uncertain in the current context, an active and dynamic educational environment should be provided with the help of appropriate and effective digital technologies to compensate for insufficient clinical competency. 

## Acknowledgement

We would like to thank the nurse educators and nursing students of Birjand School of Nursing who helped the researcher in conducting the research.

**Conflict of Interest:** The author(s) declared no conflict of interest with respect to the research, authorship, and/or publication of this article.
